# Aperiodic component of EEG power spectrum and cognitive performance are modulated by education in aging

**DOI:** 10.1038/s41598-024-66049-2

**Published:** 2024-07-02

**Authors:** Sonia Montemurro, Daniel Borek, Daniele Marinazzo, Sara Zago, Fabio Masina, Ettore Napoli, Nicola Filippini, Giorgio Arcara

**Affiliations:** 1 Department of Philosophy, Sociology, Pedagogy and Applied Psychology, FISPPA, University of Padova, Padua, Italy; 2 Department of Data-Analysis, Faculty of Psychology and Educational Sciences, Ghent University, Ghent, Belgium; 3 IRCCS San Camillo Hospital, Venice, Italy

**Keywords:** Neuroscience, Psychology, Health care

## Abstract

Recent studies have shown a growing interest in the so-called “aperiodic” component of the EEG power spectrum, which describes the overall trend of the whole spectrum with a linear or exponential function. In the field of brain aging, this aperiodic component is associated both with age-related changes and performance on cognitive tasks. This study aims to elucidate the potential role of education in moderating the relationship between resting-state EEG features (including aperiodic component) and cognitive performance in aging. N = 179 healthy participants of the “Leipzig Study for Mind–Body-Emotion Interactions” (LEMON) dataset were divided into three groups based on age and education. Older adults exhibited lower exponent, offset (i.e. measures of aperiodic component), and Individual Alpha Peak Frequency (IAPF) as compared to younger adults. Moreover, visual attention and working memory were differently associated with the aperiodic component depending on education: in older adults with high education, higher exponent predicted slower processing speed and less working memory capacity, while an opposite trend was found in those with low education. While further investigation is needed, this study shows the potential modulatory role of education in the relationship between the aperiodic component of the EEG power spectrum and aging cognition.

## Introduction

As we age, many changes occur in individuals’ behavior and cognition, worsening in memory^1^, attention span^2^, executive functions^3^, and processing speed^4^, reflecting just a few of the consequences of the natural aging process. These behavioral changes are accompanied by (and associated with) changes in the brain's structural anatomy^5,6^, metabolism^7^, and functionality^8^ which produce a significant effect on its neurophysiological activity^9^. Electroencephalography (EEG) studies on aging have shown changes in neural oscillatory activity, especially in the alpha band (8–12 Hz)^10–12^. Researchers have reported that older adults display slower alpha oscillatory activity and lower alpha power than their younger counterparts^8,13,14^. Moreover, individual alpha peak frequency (IAPF), i.e., the frequency where EEG activity exhibits the maximum power in the alpha range, tends to decrease from adulthood to midlife^11,12^. In most studies, EEG activity in specific frequency bands has been traditionally measured as the average of the power in the frequency bands of interest as calculated from the power spectrum^15^. This approach has been recently questioned by a renewed interest in the non-oscillatory, aperiodic component of the EEG signal.

The aperiodic component exhibits a 1/f-like distribution in the semi-log space of a Power Spectrum Density (PSD), meaning its power exponentially decreases as frequency increases.

Aperiodic activity can be parametrized by values of the exponent, which describes the negativity of the power spectrum slope, and the offset, the broadband shift of power across frequencies^15^. Importantly, changes in the spectrum's aperiodic component may occur without changes in the oscillatory components and may affect the power values calculated for each frequency. This may lead to spurious results and wrong interpretations when focusing only on the periodic activity and highlights the importance of taking into account the aperiodic component when interpreting power spectrum data^15,16^.

In the context of aging, it has been shown that the aperiodic slope of EEG and electrocorticography (ECoG) spectra flattened in a group of older people compared with a younger one, with decreased power between 8 and 14 Hz and increased power between 14 and 25 Hz^17^. The changes in EEG spectral slope were also associated with age differences in working memory performance. Interestingly, the aperiodic exponent appeared to mediate this relationship, suggesting that the slope effect alone could account for behavioral differences between older and young adults. Voytek and colleagues explained these results with the “Neural Noise Hypothesis”^17^, initially suggesting that as people age, there is an increase in spontaneous desynchronized neural activity, resulting in a decreased fidelity of neural communication and a flatter power spectrum. Recent studies have replicated Voytek’s findings and added new insights to the relationship between aging and aperiodic spectral components, highlighting their impact on cognitive performance. As an example, flatter slopes in older adults have been linked to poorer performance during spatial attention tasks^18,19^ and short-term memory tasks^20^. Recent work from Pathania and colleagues^21^ has associated the flattening of the aperiodic slope in frontal regions with worse performance on tasks involving processing speed and executive functions. Another study^18^ measured changes of the aperiodic spectra at the baseline period in younger and older adults investigating to what extent 1/f like exponent was related to alpha trial-by-trial consistency in a spatial discrimination task. The authors found that older adults with the highest baseline noise levels also had the worse alpha trial-by-trial consistency, suggesting that age-related increases in baseline noise might diminish sensory processing and cognitive performance.

Understanding the impact of neural noise could suggest new perspectives on the relationships between aging and neurophysiological functioning, also when considering other moderating variables^22–28^. In this field, a crucial role could be played by “Education” a variable indicating the educational level, typically operationalized as years of successful education or as an ordered factor (e.g. high school, university, etc.). Education is typically strongly associated with cognitive performance (i.e., the higher the education, the better cognitive performance), and it is almost always considered in any study on aging. The theory of Cognitive Reserve^21,23^ has suggested a causal role of Education, as it is related to further life experiences (e.g., complexity of the occupational level) that may influence the capacity of brain structure and functions to cope with age-related changes (normal and pathological).

Although some results already suggest that education is associated with changes in the aperiodic component^29^, it is not known whether education may modulate the effect of aging on spectral properties and in particular of the aperiodic component, in a possible interaction between these variables.

The present work aims to fill this gap, by investigating the potential moderating role of education on the relationship between aging and spectral properties of EEG, with focus on the aperiodic activity. To this aim, we analyzed the resting state and behavioral data of three groups: young adults with high education, older adults with higher education, and older adults with lower education. More specifically, the aims of the study were: (i) to investigate the possible age-related changes in the EEG spectral properties across these groups, (ii) to investigate the age-related cognitive differences as measured with test scores across the three groups, (iii) to investigate the role of education as a variable expected to moderate the association between EEG spectral properties and cognitive measures^25^. Older adults with higher education were expected to preserve a more youth-like profile as compared to older adults with lower education. Exploring the role of education in relationship with multiple facets of spectral properties and in particular of aperiodic component of the resting-state EEG signal might expand the research about brain aging and its impact on cognitive outcomes.

## Results

### Descriptive analyses

**Figure 1:**
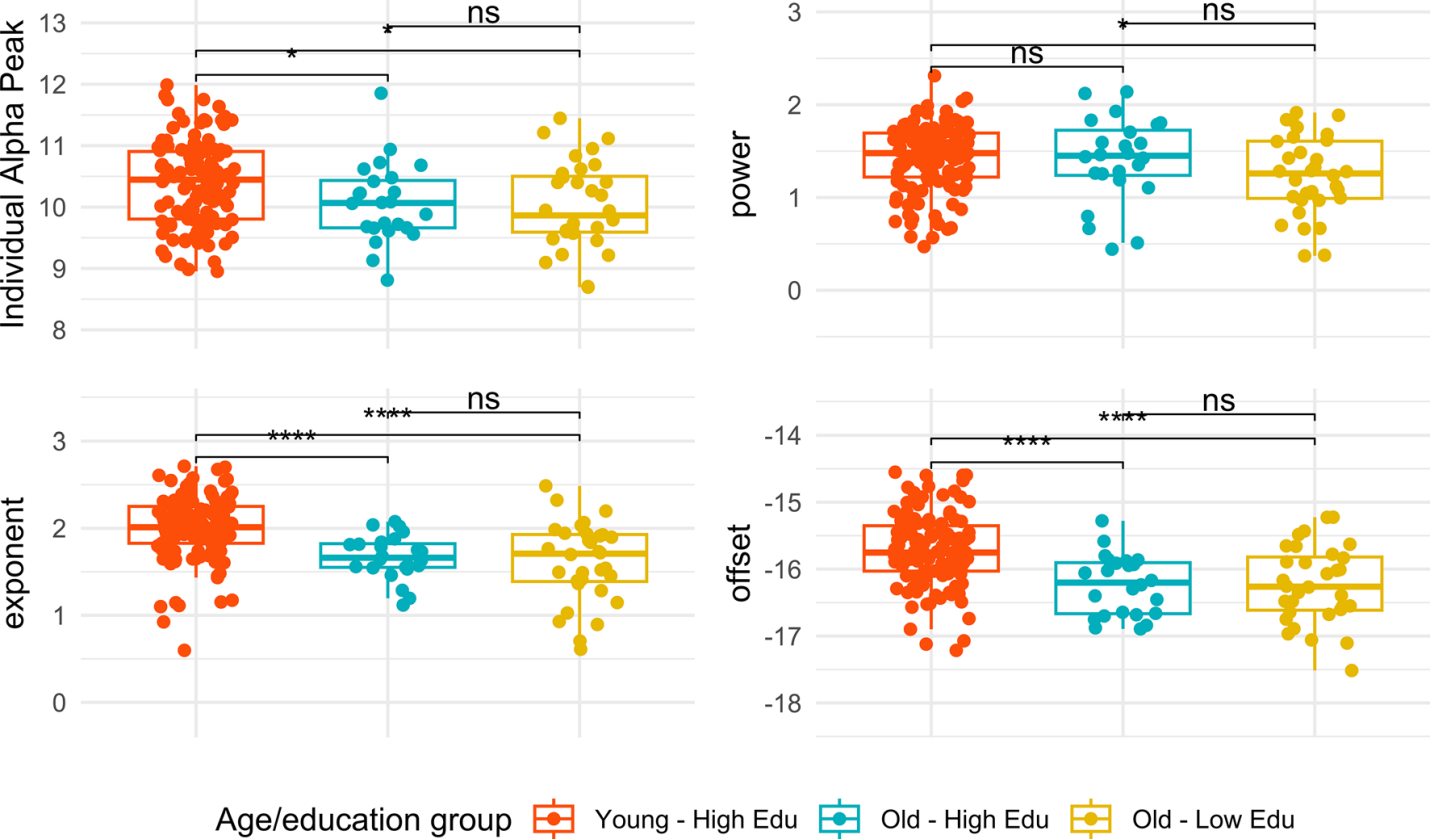
Age- and Education-related differences of the EEG components. The Individual Alpha Peak Frequency, power, exponent, and offset of each group (young – high edu = younger adults with high education, old – high edu = older adults with high education; old – low edu = older adults with low education) are shown on the y-axis.

Older adults performed significantly worse on all cognitive tasks than younger adults [Visual Attention response times (t = 9.26, p < 0.001; Cohen’s d = 1.85); Alertness response times (t = 6.66, p < 0.001; Cohen’s d = 1.25); Working Memory accuracy (t = −2.57, p < 0.001; Cohen’s d = 0.85); Delayed Memory accuracy (t = −13.46, p < 0.001; Cohen’s d = 1.11)]. IAPF, exponent, and offset values were different in young adults compared to older adults [i.e., IAPF (t = −3.23, p = 0.001; Cohen’s d = 0.48); exponent (t = −6.02, p < 0.001; Cohen’s d = 1.91); offset (t = −6.10, p < 0.001; Cohen’s d = 0.99)]; see Table S1 as Fig. 1 and Fig. S1.

Older adults with different educational levels did not differ on most cognitive tasks except for the working memory one [visual attention response times (t = −0.11, p = 0.91); alertness response times (t = −0.25, p = 0.79); working memory accuracy (t = 2.71, p < 0.01; Cohen’s d = 0.72); delayed memory accuracy (t = −1.14, p = 0.25)], where older adults with high education performed better than older adults with lower education and more similarly to the younger adults (see also Table 1).

**Table 1:** Descriptive information about the sample and group comparisons based on participants’ age and education.

	Young adults		Older adults with high education		Older adults with low education		Group comparison Young-high edu vs old-low edu		Group comparison Young-high edu vs old-low edu		Group comparison Old-low edu vs old-low edu	
	Range	M(SD)	Range	M (SD)	Range	M (SD)	*χ*^2^	*p*	*χ*^2^	*p*	*χ*^2^	*p*
PS_Allertness	(−1.69 to 2.67)	−0.43 (0.6)	(−0.33 to 3.35)	0.69 (1.07)	(−0.66 to 5.29)	0.77 (1.07)	23.15	< 0.001	30.44	< 0.001	< 0.01	0.92
PS_Visual Attention	(−1.47 to 1.50)	−0.43 (0.56)	(−0.71 to 3.62)	0.94 (1.07)	(−0.84 to 3.59)	0.97 (1.07)	42.84	< 0.001	42.11	< 0.001	0.08	0.77
MEM_WM	(−2.83 to 0.80)	0.24 (0.71)	(−3.27 to 0.80)	−0.04 (1.28)	(−3.72 to 0.80)	−0.92 (1.28)	0.64	0.42	26.30	< 0.001	8.13	< 0.01
MEM_Del	(−2.46 to 1.27)	0.30 (0.84)	(−3.51 to 0.79)	−0.85 (0.97)	(−2.46 to 1.08)	−0.54 (0.97)	24.16	< 0.001	23.20	< 0.001	0.98	0.32
GM_Volume	(0.42 to 0.50)	0.46 (0.01)	(0.36 to 0.44)	0.41 (0.01)	(0.38 to 0.45)	0.42 (0.01)	56.63	< 0.001	65.30	< 0.001	2.07	0.14
IAPF	(8.95 to 11.98)	10.38 (0.68)	(8.81 to 11.85)	10.04 (0.67)	(8.69 to 11.44)	10.00 (0.67)	3.96	0.04	5.46	0.01	0.17	0.67
Power	(8.95 to 11.98)	10.38 (0.68)	(8.81 to 11.85)	10.04 (0.67)	(8.69 to 11.44)	10.00 (0.67)	3.96	0.04	5.46	0.01	0.17	0.67
Exponent	(0.59 to 2.71)	2.00 (0.36)	(1.11 to 2.07)	1.67 (0.38)	(0.61 to 2.48)	1.61 (0.38)	22.19	< 0.001	20.77	< 0.001	0.21	0.64
Offset	(−17.21 to 14.55)	−15.72 (0.54)	(−16.89 to 15.28)	−16.23 (0.51)	(−17.52 to 15.22)	−16.24 (0.51)	18.33	< 0.001	18.61	< 0.001	0.02	0.88

Table depicts: processing speed alertness (PS_Allertness), processing speed visual attention (PS_Attention), working memory accuracy (MEM_WM) and delayed memory accuracy (MEM_Del); gray matter volume normalized (GM_Volume), individual alpha peak frequency (IAPF), exponent, and offset.

### EEG spectral parameters and cognitive performance

**Figure 2:**
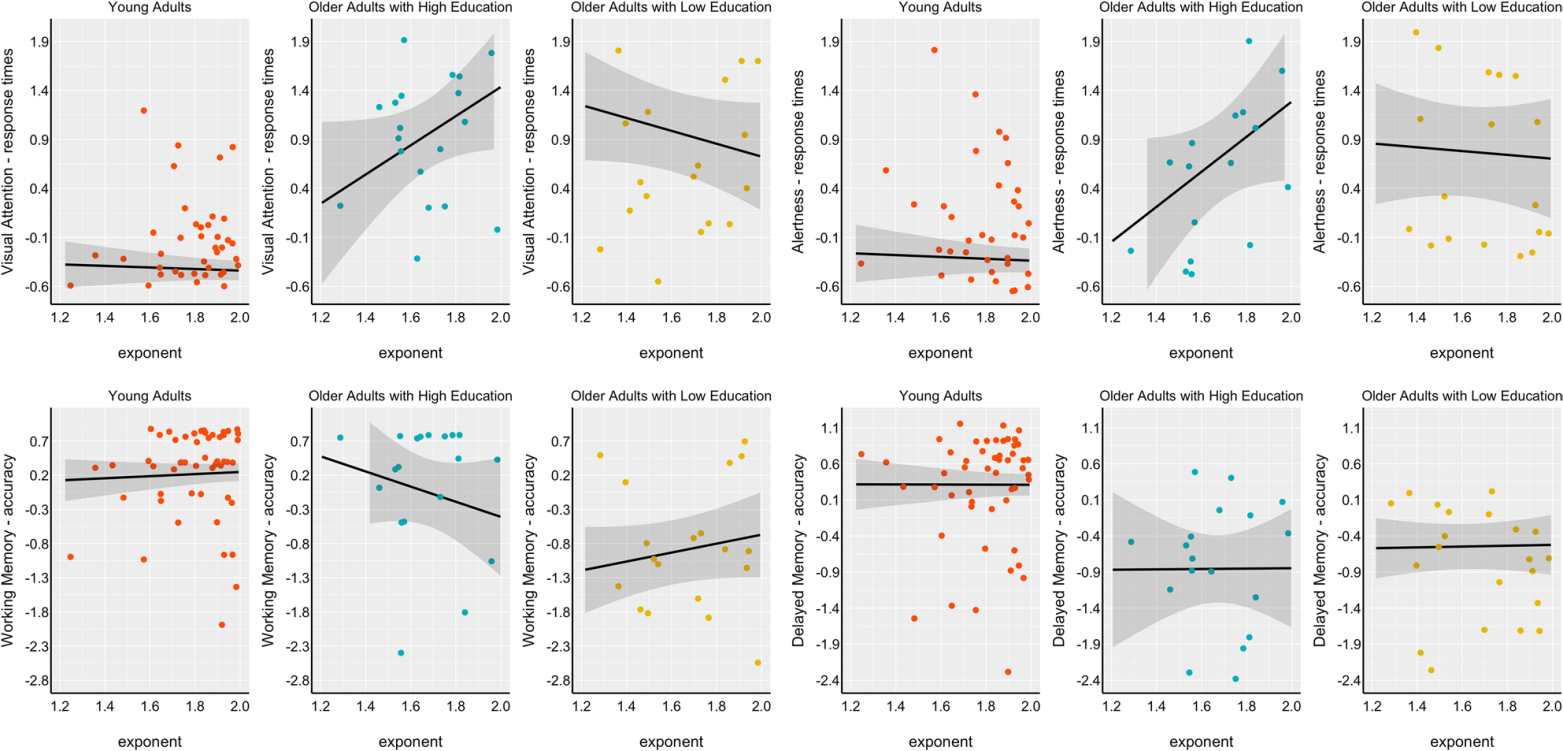
Relationship between exponent and cognitive performance on different tasks. On the upper side of the panel, response times in the visual attention and alertness tasks (processing speed) are reported on the left- and right-hand sides respectively. On the lower panel, accuracy in the working and delayed memory tasks are reported on the left- and right-hand sides respectively. On the x-axis, the exponent value parameterized at the occipital level and in the Alpha band (8–12 Hz) is reported. On the y-axis, the z-scores associated with the task outcome.

In the whole sample, a higher exponent and offset significantly predicted a better performance on the visual attention task, i.e., a faster performance in terms of response time [(exponent: *B* = −0.44, *p* < 0.01; Cohen’s f^2^ = 25.11); (offset: *B* = −0.32, *p* < 0.01; Cohen’s f^2^ = 25.63)]. The exponent and the offset values predicted better working memory capacity in terms of response accuracy [(exponent: *B* = 0.37, *p* = 0.04; Cohen’s f^2^ = 6.91); (offset: *B* = 0.31, *p* = 0.01, Cohen’s f^2^ = 7.96)]. Significant results emerged when considering the three groups, i.e., young adults, older adults with low education, and older adults with high education, both for the exponent and the offset, in the visual attention and working memory tasks. Compared to the group of young adults, where exponent and offset did not predict any variation in cognitive performance, a significant interaction was shown in older adults depending on their educational level. In the visual attention task, low-educated older adults had a better (faster) performance at the higher aperiodic values [(exponent: *B* = −0.67, *p* = 0.04; Cohen’s f^2^ = 22; (offset: *B* = −0.56, *p* = 0.03, Cohen’s f^2^ = 21)] while those highly educated had a worse performance (slower) at the higher values of the exponent (exponent: *B* = 1.41, *p* = 0.02; Cohen’s f^2^ = 22), a result that was confirmed post-hoc through slope comparisons, showing a general age-related effect and also a significant difference between older adults with different education, at the third quartile of the exponent values (t = 2.45; p = 0.03), see Fig. 2.

Results showed that also in the working memory task, older adults with high education had worse performance with increasing exponent values, as compared with those with low education (*B* = −1.71, *p* = 0.03, Cohen’s f^2^ = 7.25). Post-hoc slope comparison showed no main education-related difference among older adults. Young adults and highly educated older adults did not differ from each other at different quartiles of exponent values (25%: t = 1.16, p = 0.47; 50%: t = 1.94, p = 0.12; 75%: t = 2.12, p = 0.08).

An additional exploratory analysis in a frontal ROI^29^ showed that a significant interaction also emerged at the level of the alertness task (B = 2.01, p = 0.01, Cohen’s f^2^ = 9) with higher power predicting faster response times in older adults with high education.

## Discussion

The present study aimed to investigate the relationship between aperiodic activity and cognitive performance, by accounting for the level of education in older individuals, compared with a control group of highly educated younger adults (i.e., high neurocognitive efficiency).

The older adults showed less cognitive efficiency compared to young adults, across all tasks, which aligns with the well-established literature about cognitive decline in healthy aging^30^. Upon considering older adults stratified based on education, the results indicated that those with higher education exhibited comparable performance to older adults with lower education, except for working memory performance. Older adults with higher education showed better working memory performance compared with older adults with lower education, which made the group of older adults with higher education more similar to the young group and suggesting a potential role of education in mitigating age-related cognitive decline, at least for some specific cognitive functions or tasks.

Consistent with previous evidence^17,31,32^, the periodic and aperiodic components of EEG differentiated between young and older adults: older adults exhibited lower values across these components, compared to younger adults, except for the parametrized power. Concerning the periodic component of the EEG signal, results showed a pattern of an age-related slowing of IAPF, reflecting findings related with previous studies^33,34^. Several interpretations have been advocated to link this periodic EEG components with aging. In addition to slowing with age, structural alterations in the brain have also been associated with the decline in power and peak frequency of alpha oscillations, particularly in older individuals^10,35^.

Additionally, the stability of power and IAPF over the life course reflects the preserved functionality of the central nervous system^36^. Regarding cognition, IAPF has previously demonstrated a positive relationship with interference resolution in working memory performance, primarily observed in the temporal lobes^29^. Our results indicate that, at least at the level of frontal brain areas (as it possible to observe in Supplementary Materials), power may play a functional role in the ability to sustain alertness and to disregard and suppress interfering information.

In relation to the aperiodic EEG components, we also found that both exponent and offset values significantly decreased with age. These results corroborate previous evidence suggesting that the aperiodic EEG component can serve as a neurophysiological marker of aging. Likewise, recent studies have revealed that aperiodic activity is influenced by various factors, including drugs^37,38^ and level of arousal^39^. However, the potential mediation of education, and more specifically its influence on the relationship between aperiodic components and cognitive performance, was unexplored in the literature.

In our study, education might help in interpreting the relationship between the aperiodic component and performance on some tasks of visual attention and working memory, but not on a delayed memory task. The relation between aperiodic component and cognitive performance varied depending on education level, with a reversed pattern between exponent and cognitive performance in older adults across higher vs lower education. Older adults with lower education displayed a positive relationship between exponent and cognitive performance, while those with higher education exhibited the opposite trend. In this context, research evidence suggests that low exponent values (when the exponent approximates zero) may reflect an increase in asynchronous background neuronal firing, commonly called neural noise^32^.

Related to the concept of neural noise, in non-linear systems like the brain, the notion of stochastic resonance proposes that information at the threshold level can be better processed within an optimal noise range than without noise^40^. If different exponent values represent varying levels of neural noise, it is possible that noise also has different effects on performance according to a specific system. In older adults with lower education levels, higher exponents—corresponding to lower noise values—may contribute to better performance. On the other hand, older adults with higher education would exhibit the opposite pattern. In this latter group, higher exponents (lower noise values) would reduce performance efficiency. These two scenarios may depend on the fact that, according to the framework of stochastic resonance, there is no ideal level of noise and its effect on performance may not follow a linear pattern: it can vary based on the specific system and compensatory dynamics. Although such a result may seem counterintuitive, a similar reversed pattern has been observed in a previous study that examined the relationship between mathematical achievement and glutamate concentrations. Glutamate has the effect of flattening the power spectrum, leading to exponent values closer to zero^37^. In a previous study^41^, it was demonstrated that the concentration of glutamate and the exponent levels could result in reversed cognitive performance outcomes depending on the participants' age. Specifically, the authors found that the concentration of glutamate (in the intraparietal sulcus) was negatively associated with mathematical achievement in younger participants, but it was positively associated with mathematical achievement in older participants. Given a possible relationship between glutamate and exponent levels^37^, these findings may be interpreted as follows: while in younger participants high levels of noise (corresponding to a lower level of glutamate and, consequently, higher exponent values) may reduce performance, in older participants high levels of noise may lead to an opposite effect, contributing to improving cognitive performance. In summary, these results, similar to ours, imply that the relationship between exponent, noise, and cognitive performance may not be straightforward, highlighting the importance of investigating possible mediators, such as education, within this complex relationship.

While tantalizing, considering education as a possible mediator in the relationship between the aperiodic component and cognitive performance may present pitfalls because education may introduce several other aspects that affect performance differently. For example, education may impact cognitive strategies, task engagement, and compensatory mechanisms, leading individuals with higher education to have a better cognitive performance with different potential explanations of the observed effect in the aperiodic EEG component. A more comprehensive definition about how this effect can be attributed to potential compensation mechanism could require further investigations.

We did observe a relationship between the exponent and processing speed, in line with a previous study^42^. Moreover, the results of the present study partially replicated what was found in some previous studies where a relationship between exponent and working memory performance was identified^15,17^. The lack of effects of aperiodic component in delayed memory task performance is of interest, as it suggests that the modulating role of aperiodic component may not be non-specific and happening for general cognitive functioning, but only for some aspects of cognitive functioning, possibly related to those case in which there is much time pressure in cognitive performance (as psychomotor or working memory tasks).

Overall, our results cannot be interpreted as exhaustive; they should emphasize the importance of considering the aperiodic component of EEG signal as a marker of neurophysiological mechanisms that relate to performance in some cognitive tasks, which can be mediated by different aspects. Our study, in particular, focused on education as one of these aspects. An important limitation is related to the fact that the LEMON database, despite having many advantages, did not have the optimal characteristics for the aims of this study. In particular, it included a cohort of participants with different ages (whereas a longitudinal dataset would have been more suited) and it included a different number of participants for each group, with a larger size for the group of younger adults as compared to the older adults. In this study, we focused on the occipital ROI. This decision was based both on previous scientific literature which led to the expectation of dominant age-related patterns in the alpha domain^8,10,12–14^ and to the limited spatial resolution of high-density EEG in which electrode localization data were only partially available for this dataset^43^. The additional exploratory analysis on frontal brain regions supported the potential interaction between EEG measures, education and cognitive performance that needs to be further explored through techniques with better spatial resolution^44^.

Future studies with better stratification might explore the ontogenetic trajectory of the exponent, to further investigate its role in cognitive performance across different tasks during aging. In fact, in the present study, the availability of age and education variables in a categorical form might have limited the assessment of neurobehavioral relationships and the potential use of finer analysis modeling (i.e. age was included as a factor rather than a continuous variable).

Future studies may explore the intricate connection between EEG parameters and cognition, by encompassing a broader range of variables that could modulate such a relationship, such as life experience variables, or others associated with physical health and physical activity, or to other proxies that can be traced back to the concept of “cognitive reserve”, which may be crucial in understanding the complex relationship between cognitive and brain aging. Finally, it is important to stress a limitation (intrinsic to cross-sectional and quasi-experimental studies), that is the impossibility to infer cause-effect relationship. In all cases, the associations observed between EEG spectral parameters and performance should not be interpreted as evidence of causal relationships, but rather as a statistical association in which the directionality is not known and that could be mediated also by other aspects.

In summary, results from this study opens many question that may guide future research on the modulatory role of education and other cognitive reserve proxies, in the complex relationship between aperiodic EEG component and cognitive efficiency in aging.

## Methods

### Participants and materials

All participants included in this study were taken from the “Leipzig Study for Mind–Body-Emotion Interactions'' (LEMON^43^). The final sample consisted of N = 179 individuals. Socio-demographic information like age and education was shared in bins^43^, not continuous. In the LEMON project, two groups are distinguished: one group of young adults and another of older adults. Such groups were maintained in our study, also based on previous research using a similar approach^45,46^.

Participants included in the young group (N = 123) aged 20–35 years and all had high education levels (12 years of *lyceum/gymnasium*), whereas the group of older adults (N = 56), age range 60–77 years, was divided into two groups: one with high education (12 years of *lyceum/*gymnasium, N = 24) and the other with low education (10 years of *technical high school/Realschule*, N = 32)*.* Participants with no availability of EEG data and those who were indicated as with alcohol abuse or dyslexia problems were not included in the final data sample. A small subgroup of young adults with low education was not included in the sample according to the study purpose (N = 7); one participant resulted as an outlier on both visual inspection of residuals and Cook's threshold, and it was removed. For this study, data collection was performed in accordance with the Declaration of Helsinki; it was approved by the ethics committee, reference number 154/13-ff (University of Leipzig) where all participants provided their written informed consent prior to data acquisition for the study, including their agreement to their data for being shared anonymously (for more details please consider the article of Babayan and colleagues)^43^.

### Cognitive assessment

Processing Speed and Memory capacity were investigated in relationship with periodic and aperiodic EEG components. From the LEMON dataset, we adopted those tests that have been mostly used in the literature to evaluate cognitive impairment in older adults in relationship with the periodic and aperiodic components of the EEG power spectral density^29,47^.

- Processing speed included alertness and visual attention and it was assessed using two tasks: the Test of Attentional Performance^48^ and part B of the Trail Making Test (TMT)^49^. The former estimated alertness: i.e., participants were asked to respond, as fast as possible, to the appearance of a visual stimulus on the screen. The TMT-B estimated visual attention, i.e., participants were asked to connect as fast as possible a series of visual stimuli, alternatively with a definition order: numerical and alphabetical orders. In particular, the time of completion of the task was recorded.
- Memory included working and delayed memory tasks; it was assessed with a working memory task (WM_TAP)^48^ and the California Verbal Learning Task (CVLT)^50^. For the working memory task, participants had to simultaneously provide a response only when a given stimulus was equal to the second last one perceived in the series while keeping track of a series of different stimuli. In the delayed memory task, participants were asked to retain and correctly recall a series of 16 words belonging to their vocabulary.

### Neural variables

#### Gray matter volume

A 3 Tesla scanner (MAGNETOM Verio, Siemens Healthcare GmbH, Erlangen, Germany) with a 32-channel head coil was used to conduct Magnetic Resonance Imaging (MRI)^43^. The pre-processing pipeline included a series of steps: (a) re-orientating images to the standard (MNI) template, (b) bias field correction, (c) registration to the MNI template using both linear (FLIRT) and nonlinear (FNIRT) registration tools, and (d) brain extraction. Brain tissues were segmented using FMRIB's Automated Segmentation Tool (FAST) which allowed extracting measures of total Gray Matter, White Matter, and Cerebrospinal Fluid. Brain tissues were visually inspected by a trained neuroscientist (NF) to ensure an accurate segmentation.

#### EEG preprocessing and source reconstruction

The eyes-closed resting-state EEG recording (8 min) present in the LEMON project was analyzed^43^. The recording was made with a BrainAmp MR plus amplifier in an electrically shielded and sound-attenuated EEG booth using 62-channel (61 scalp electrodes plus 1 electrode recording the VEOG below the right eye) active ActiCAP electrodes (both Brain Products GmbH, Gilching, Germany), referenced to FCz. EEG was recorded with a band-pass filter between 0.015 Hz and 1 kHz and digitized with a sampling rate of 2500 Hz. Raw EEG data were down-sampled from 2500 to 250 Hz and band-pass filtered within 1–45 Hz. Outlier channels were rejected after visual inspection for frequent jumps/shifts in voltage and poor signal quality. Data intervals containing extreme peak-to-peak deflections or large bursts of high-frequency activity were identified by visual inspection and removed. Independent component analysis (ICA) was performed using the Infomax algorithm (*runica* function from MATLAB). On pre-processed files, source reconstruction was run by using a standard head model. A 3-shell boundary element model was constructed via Brainstorm^51^. The default current density maps were normalized through the Standardized LOw Resolution brain Electromagnetic TomogrAphy approach (sLORETA)^52^. Welch's method was used to calculate the power spectrum at the level of the reconstructed sources; the window size was 1 s and the window overlap was 50%. Due to the small number of EEG channels, we grouped cortical vertices into major regions (ROIs), aggregated according to Desikan-Killiany atlas following a similar approach previously used^29^ (Table S10).

#### Periodic and aperiodic components of the power spectral density

The specparam algorithm (version 1.0.0)^15^ was used to parametrise power spectra of ROIs. In specparam algorithm, the power spectrum $PSD$ is modeled as a combination of the aperiodic component $AP$ and a sum of N oscillatory peaks modeled with a Gaussian:

$$P=AP+{\sum\limits_{n=0}^{N}{G}_{n}}.$$

The component $AP\left(f\right)$ for frequency $f$ is expressed by the formula:

$$AP\left(f\right)=b-log\left(k+{f}^{\chi }\right),$$

where $b$ is the broadband offset, $\chi$ is the exponent and $k$ is the knee parameter, controlling the “bend”. When $k=0$ the component $AP$ will be a line fitted in the log–log space (this is later referred to as a fixed mode). In this case, the slope of the line $a$ in log–log space is directly related to the exponent $\chi$ , $\chi =-a$^15^. The outputs of the algorithm for estimated peaks are the mean of the Gaussian ${G}_{n}$ for the center frequency of the peak, aperiodic-adjusted power (the distance between the peak of the Gaussian and the aperiodic fit at this frequency) and bandwidth as 2 *SD* of the fitted Gaussian.

In the current analysis, power spectra were parameterized across the frequency range from 3 to 48 Hz (the maximal frequency range to avoid the line noise frequency) using the “fixed” mode. Additional algorithm settings were set as: peak width limits: [2.5 8]; max number of peaks: 6; minimum peak height: 0.05; peak threshold: 2. All the parameters describing identified peaks, offset, exponent, and the parameters describing how well the model was fit were extracted.

The parameters were extracted for every PSDs in the eyes closed condition.

As we did not have specific expectations on the pattern of spatial distribution we focused on an occiptal ROI that included parcels from Desikan-Killiany atlas which is where dominant activity in alpha is expected to exhibit age-related patterns based on a wide amount of previous literature in the context of aging^33,34,36^. The parameters from all ROIs belonging to this region were averaged. The choice of parameters gave a median goodness-of-fit measure of ${r}^{2}$ = 0.981, IQR = [0.971, 0.978] across all regions within the occipital lobe aggregated accordingly to Desikan-Killiany atlas following a similar approach previously used^29^. An additional exploratory analysis, not initially planned, was also added on a frontal ROI using other parcels from Desikan-Killiany Atlas, similar to a previous study^29^ (see Supplementary Materials for details, Table S10).

Model fits were not statistically different between the two groups: young adults (YA) median r^2^ = 0.983, IQR = [0.974, 0.988]; older adults (OA) median r^2^ = 0.976, IQR = [0.962, 0.984]. Thus, although other processing parameters could have been chosen, we achieved suitable spectral parameterization across participants and regions. Compared to previous studies using the specparam algorithm, where the frequency range varies, many used 40 Hz as the upper frequency range^29,53,54^. For the sake of clarity, while the preprocessed data shared by the LEMON consortium was filtered with a cut-off at 45 Hz before source reconstruction, our setting for spectral parameterization used 48 Hz as the upper limit frequency. We choose this latter value for three main reasons: first, it is a widely adopted option^55,56^; second, it avoids biased results due to filter roll-off effect, and third, it is in line with the recommendations of the authors of *specparam* algorithm^15^. Importantly, the use of the two upper limits for the band-pass filter (45 Hz or 48 Hz) lead to negligible differences on results and statistical significance (in Supplementary Materials).

**Figure 3:**
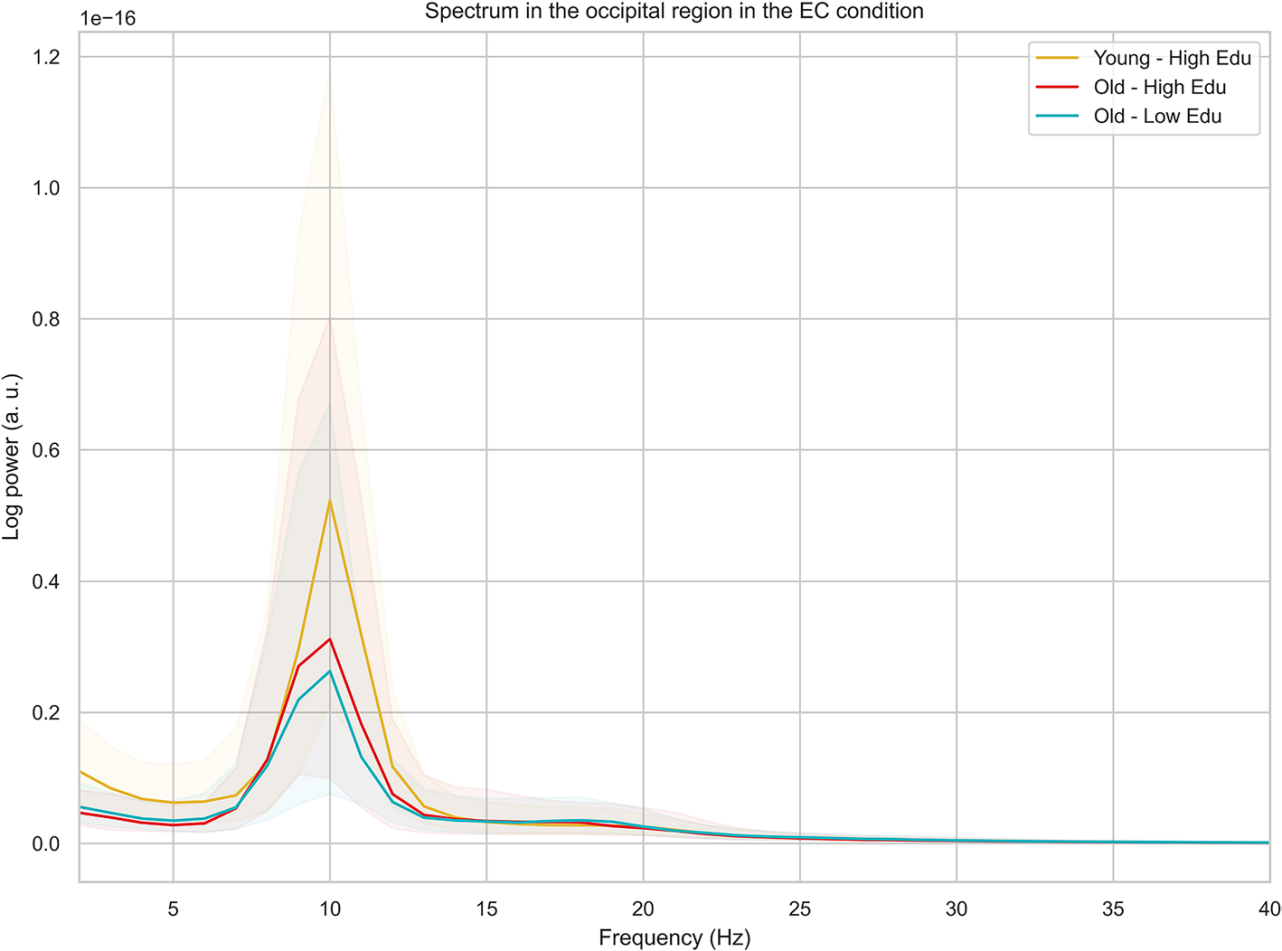
Age- and education-related differences in resting EEG power spectra in the occipital region, in the eyes closed condition. The plot shows a median for each group (young-high edu = younger adults with high education, old-high edu = older adults with high education; old-low edu = older adults with low education) and a 50% percentile interval, ranging from the 25 to 75 percentiles.

All participants showed a discernible alpha peak in the PSD (see an example in Fig. S2). Individual alpha peak frequency values per subject were estimated using periodic components fitted by the algorithm in the alpha range. IAPF was computed by analyzing the peak frequency within that range that exhibits the highest power spectral density (measured by the value of power of the peak, in the individual's EEG data per ROI and then averaged across ROIs within the occipital region. This method is preferred over averaging frequencies of all peaks as it identifies the dominant oscillatory rhythm of the individual's brain activity, providing a more accurate marker for cognitive and attentional processes^12,13,57^. Figure 3 offers a qualitative overview of the differences in EEG power spectra among our groups. The boxplots in Fig. 1 visually confirmed the differences in IAPF (Individual Alpha Peak Frequency) values between the young and old populations.

### Statistical analyses

Analyses were performed with the R software^58^. A correlation matrix (Spearman’s method) was used to show the pattern of correlation among variables of interest (Figure S3). Visual inspection of distribution of variables, Shapiro–Wilk tests on residuals, and Kolmogorov–Smirnov analysis were performed prior to build up the regression models. The results of these analyses indicated General Linear regression Models as suitable; they included processing speed and accuracy scores on cognitive tests as dependent variables transformed in z-scores^29^, and the variable group as a factor: older adults with high education vs. older adults with lower education vs. young adults (all high education). The continuous predictors were the periodic and the aperiodic EEG components: IAPF, power, exponent, and offset values. Sex and normalized gray matter volume were accounted for in all regression models. The regression model analyses have been carried out on Occipital ROI. An additional exploratory analysis was carried out also on a Frontal ROI, which can also be vulnerable in aging individuals^44^.

A simplified syntax of the R linear models is reported below:

$$test \, score \, \sim \, EEG \, parameter \, x \, group \, + \, sex \, + \, Gray \, Matter \, volume$$

Sex and Gray matter volume were added as covariates as they were two relevant variables that could also be associated with cognitive performance. Power analysis revealed a statistical power greater than 0.95, indicating the ability of the model to detect significant effects, based on a significance level (α) of 0.05 and an estimated effect size f^2^ of 0.35. Cohen's d was used for estimating the effect size in group comparisons; Cohen's f^2^ was used for the regression analyses as it took into consideration the explained variation and residual variability in the model.

### Supplementary Information

Supplementary Information.

## Data Availability

Data are available at: https://www.nature.com/articles/sdata2018308.
