# Supplementary material for: Aperiodic component of EEG power spectrum and cognitive performance are modulated by education in aging
Source: Sci Rep. 2024 Jul 2;14:15111. doi: 10.1038/s41598-024-66049-2 (PMC11220063; doi:10.1038/s41598-024-66049-2)
Supplement: Supplementary file 1 — Supplementary Information. [file 41598_2024_66049_MOESM1_ESM.pdf]

SUPPLEMENTARY MATERIALS

Aperiodic component of EEG power spectrum and cognitive performance are modulated by education in aging

Sonia Montemurro[1^*], Daniel Borek[2^], Daniele Marinazzo[2], Sara Zago[1], Fabio Masina[1], Ettore Napoli[1], Nicola Filippini[1], Giorgio Arcara[1]

[1]*IRCCS San Camillo Hospital, Venice (Italy)*

[2]*Department of Data-Analysis, Faculty of Psychology and Educational Sciences, Ghent University, Belgium*

^These authors share the first authorship

***Corresponding Author**: Sonia Montemurro, *IRCCS San Camillo Hospital, Venice (Italy)*. E-mail: sonia.montemurro@unipd.it

GROUP-DIFFERENCES

Younger adults were overall more efficient in performing cognitive tasks than older adults with both high and lower education, except in the case of working memory tasks, where older adults with higher education did not differ from younger adults.

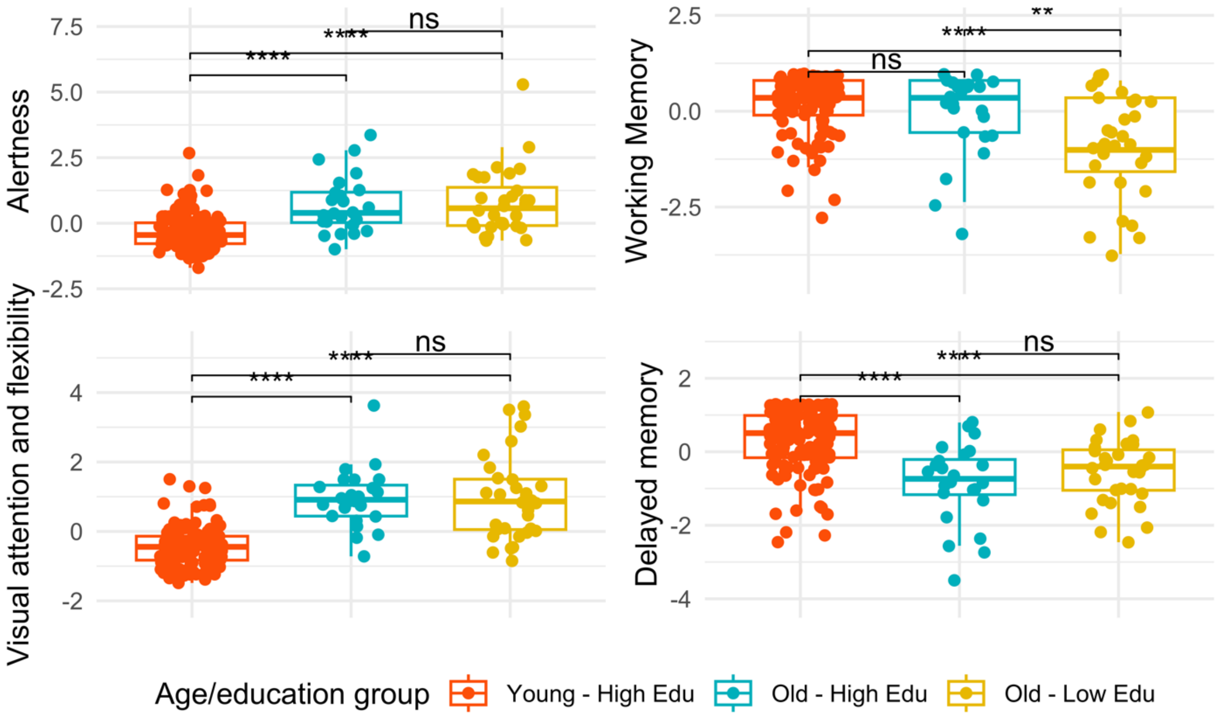

Figure S1. Group Comparisons of cognitive variables.
The panel shows, on the upper left-hand side the group-differences associated with the Alertness processing speed, on the upper right-hand side the group differences associated with the Working Memory accuracy; on the lower left-hand side, the differences associated with Visual Attention processing speed; on the lower right-hand side with Delayed Memory accuracy. Significance is set with $p<0.05$.

POWER SPECTRUM WITH APERIODIC COMPONENT

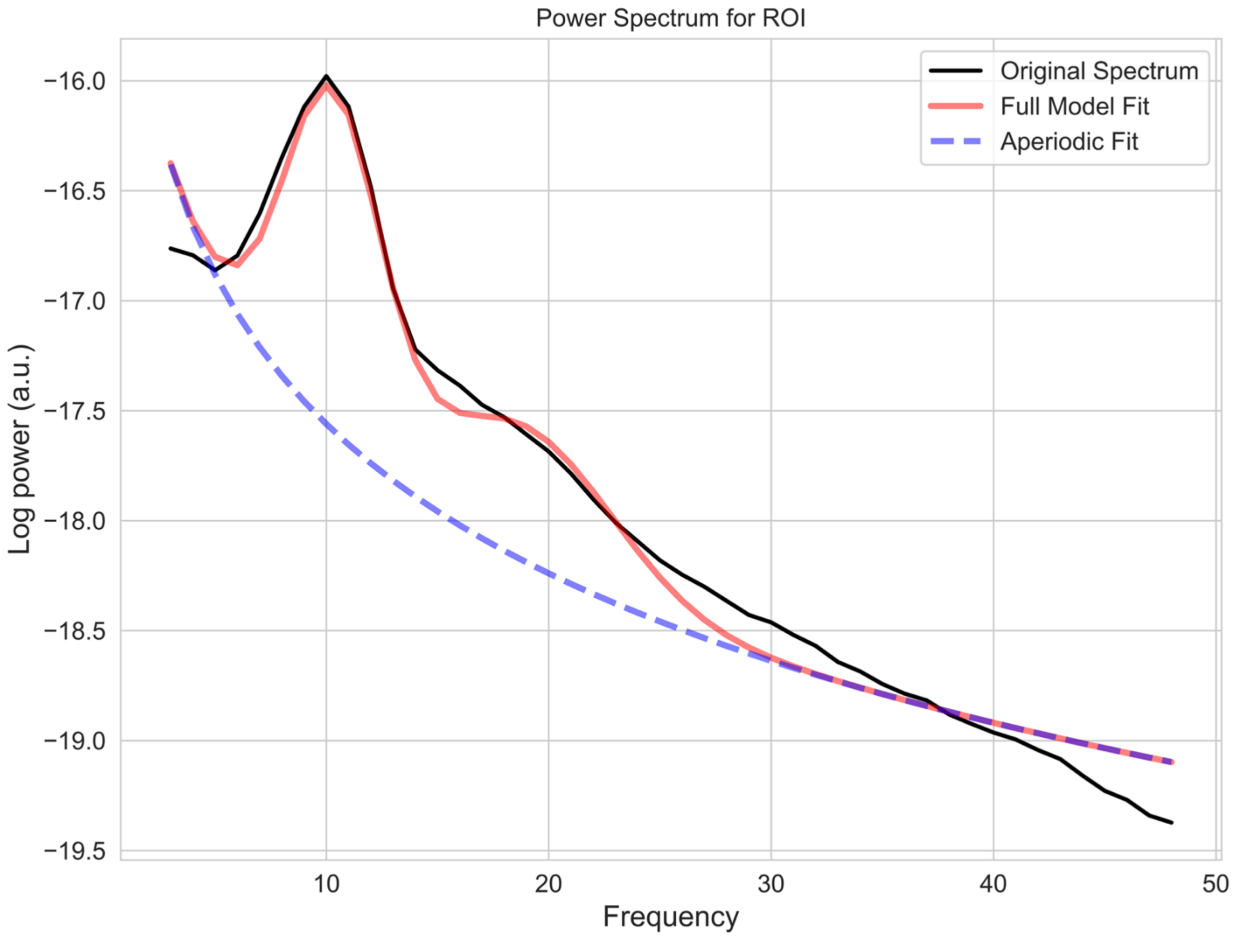

Figure S2. Example plot with fitted parameters (associated with participant 'sub-032302' right cuneus). The x-axis shows the frequency band in Hz units. The y-axis shows power in arbitrary units (a.u.) associated with resting-state activity in the eyes-closed condition at the right cuneus ROI (referred to the Desikan-Killiany atlas) for this participant.

Correlation Matrix

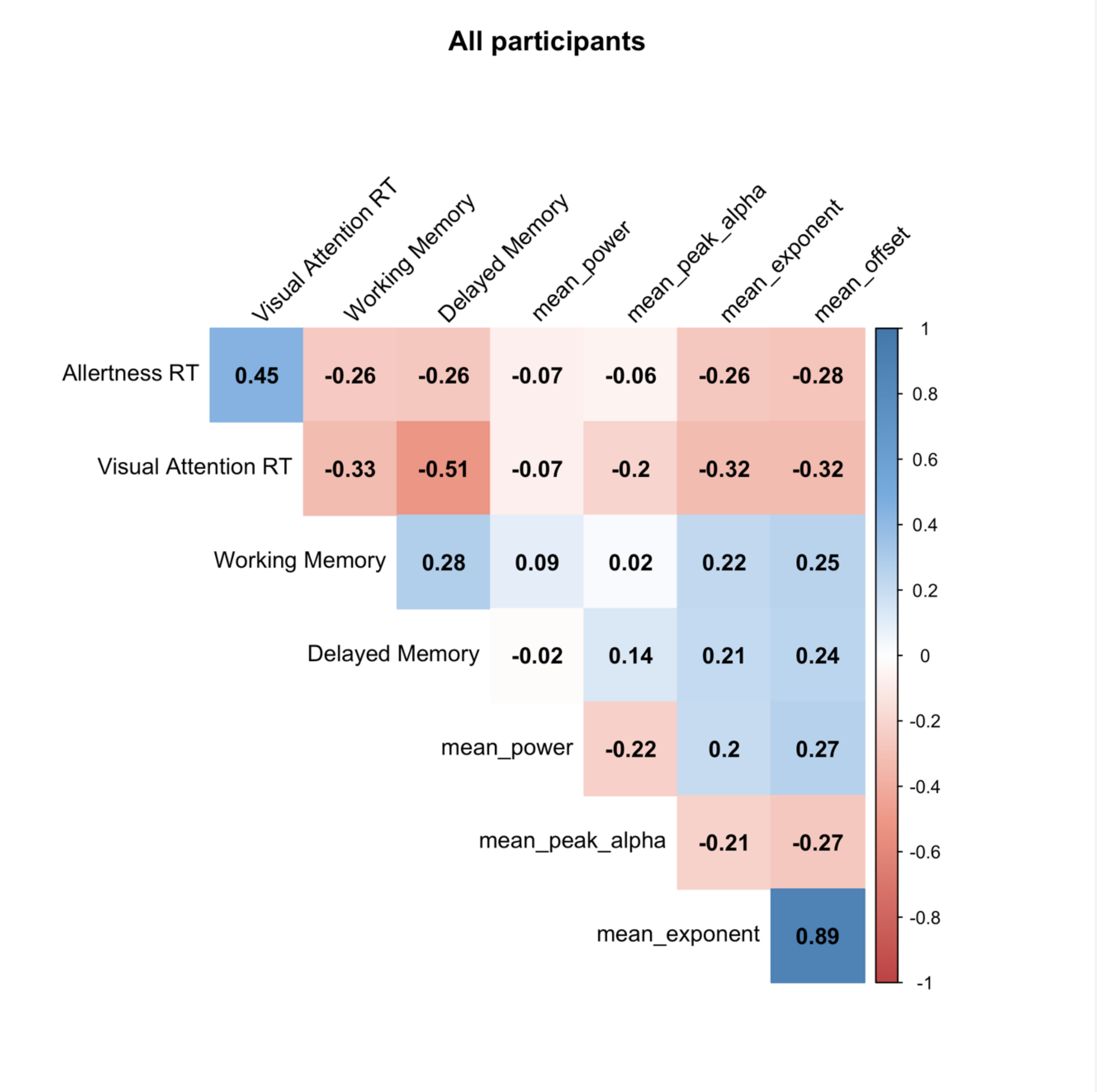

Figure S3. Correlation matrix with Spearman's Method.
The matrix reports Alertness and Visual Attention Response Times, Working and Delayed Memory accuracy, Individual Alpha Peak Frequency, power, exponent, and offset.

	Young Adults (38 F)		Older Adults (26 F)		Young - Old
	Range	M(SD)	Range	M (SD)	*p*
**PS_Attention**	(-1.47 - 1.50)	-0.43 (0.56)	(-0.84 - 3.63)	0.97 (1.07)	<0.001
**PS_Allertness**	(-1.69 - 2.68)	-0.33 (0.68)	(-0.98 - 5.30)	-0.74 (1.17)	<0.001
**MEM_WM**	(-2.83 - 0.80)	0.24 (0.71)	(-3.73 - 0.80)	-0.55 (1.28)	<0.001
**MEM_Del**	(-2.46 - 1.27)	0.30 (0.84)	(-3.52 - 1.08)	-0.68 (0.97)	<0.001
**GM_Volume**	(0.42 - 0.50)	0.46 (0.01)	(0.36 - 0.45)	0.41 (0.01)	<0.001
**IAPF**	(8.95 - 11.98)	10.38 (0.68)	(8.69 - 11.85)	10.03 (0.67)	<0.01
**Power**	(0.56 - 2.31)	1.43 (0.34)	(0.37 - 2.13)	1.31 (0.44)	0.06
**Exponent**	(0.59 - 2.71)	2.00 (0.36)	(0.61 - 2.48)	1.63 (0.38)	<0.001
**Offset**	(-17.21 - 14.55)	-15.72 (0.54)	(-17.52 - 15.22)	-16.24 (0.51)	<0.001

Table S1. Descriptive information about the sample, based on participants' age.
Age-related group comparisons on Processing Speed alertness (PS_Allertness), Processing Speed Visual Attention (PS_Attention), Working Memory accuracy (MEM_WM) and Delayed Memory accuracy (MEM_Del). Brain measures reported are gray matter volume normalized (GM_Volume), Individual Alpha Peak Frequency (IAPF), Exponent, and Offset.

Regression analyses
Occipital Brain Region

Processing Speed: Alertness		Estimate	Std. Error	t-value	Pr(>|t|)
IAPF	(Intercept)	-0,27	2,82	-0,10	0,92
	GM_Normalised	-3,23	3,95	-0,82	0,42
	sexM	-0,11	0,15	-0,77	0,44
	Young – High Edu	1,20	2,53	0,47	0,64
	Old – High Edu	3,17	3,66	0,87	0,39
	mean_peak_alpha	0,25	0,22	1,11	0,27
	Young – High Edu: IAPF	-0,22	0,25	-0,86	0,39
	Old – High Edu: IAPF	-0,32	0,36	-0,89	0,38
PW	(Intercept)	2,95	1,77	1,67	0,10
	GM_Normalised	-3,56	3,95	-0,90	0,37
	sexM	-0,11	0,14	-0,80	0,43
	Young – High Edu	-1,40	0,61	-2,29	0,02
	Old – High Edu	-0,66	0,77	-0,86	0,39
	mean_power	-0,50	0,37	-1,36	0,18
	Young – High Edu: PW	0,40	0,43	0,91	0,36
	Old – High Edu: PW	0,48	0,54	0,88	0,38
exponent	(Intercept)	3,23	1,82	1,77	0,08
	GM_Normalised	-4,55	3,97	-1,15	0,25
	sexM	-0,10	0,14	-0,70	0,48
	Young – High Edu	-1,20	0,73	-1,64	0,10
	Old – High Edu	-2,56	1,36	-1,89	0,06
	mean_exponent	-0,30	0,34	-0,89	0,38
	Young – High Edu: exponent	0,21	0,40	0,53	0,59
	Old – High Edu: exponent	1,49	0,80	1,86	0,06
offset	(Intercept)	-1,32	4,66	-0,28	0,78
	GM_Normalised	-3,62	3,99	-0,91	0,37

**Processing Speed: Alertness**		Estimate	Std. Error	t-value	Pr(>|t|)
	sexM	-0,14	0,15	-0,93	0,36
	Young – High Edu	0,89	5,00	0,18	0,86
	Old – High Edu	4,67	8,02	0,58	0,56
	mean_offset	-0,23	0,28	-0,82	0,41
	Young – High Edu: offset	0,11	0,31	0,35	0,73
	Old – High Edu: offset	0,29	0,49	0,59	0,56

Table S2. Summary output of the Regression model with alertness response times as dependent variable (Occipital ROI). The table displays, in the first column, Individual Alpha Peak Frequency (IAPF), exponent, and offset; in the second column, the terms of the mode. The third, fourth, and fifth columns refer to the B (estimate) values, the relative standard error, the t-value associated with the fitted term, and the p-value.

Processing Speed: Visual Attention		Estimate	Std. Error	t-value	Pr(>|t|)
IAPF	(Intercept)	2,16	2,46	0,88	0,38
	GM_Normalised	0,52	3,44	0,15	0,88
	sexM	0,21	0,13	1,61	0,11
	Young – High Edu H	-2,69	2,21	-1,22	0,23
	Old – High Edu	-0,53	3,20	-0,17	0,87
	mean_peak_alpha	-0,15	0,19	-0,76	0,45
	Young – High Edu: IAPF	0,12	0,22	0,55	0,58
	Old – High Edu: IAPF	0,04	0,32	0,14	0,89
PW	(Intercept)	1,08	1,54	0,70	0,49
	GM_Normalised	0,56	3,45	0,16	0,87
	sexM	0,22	0,13	1,76	0,08
	Young – High Edu	-1,93	0,53	-3,62	<0,001
	Old – High Edu	-0,33	0,67	-0,49	0,63
	mean_power	-0,34	0,32	-1,06	0,29
	Young – High Edu: PW	0,34	0,38	0,90	0,37
	Old – High Edu: PW	0,21	0,47	0,43	0,66
exponent	(Intercept)	0,07	1,64	0,04	0,97
	GM_Normalised	-1,06	3,38	-0,31	0,75
	sexM	0,24	0,12	1,95	0,05
	Young – High Edu	2,50	0,62	4,01	<0,001
	Old – High Edu	-1,06	1,14	-0,93	0,35
	mean_exponent	-0,09	0,18	-0,50	0,62
	Young – High Edu: exponent	-0,68	0,34	-1,98	0,05
	Old – High Edu: exponent	1,41	0,64	2,21	0,03
offset	(Intercept)	-1,42	2,50	-0,57	0,57
	GM_Normalised	-0,33	3,41	-0,10	0,92
	sexM	0,20	0,12	1,57	0,12

Processing Speed: Visual Attention		Estimate	Std. Error	t-value	Pr(>|t|)
	Young – High Edu	-7,72	4,28	-1,81	0,07
	Old – High Edu	6,09	5,95	1,03	0,31
	mean_offset	-0,06	0,12	-0,53	0,60
	Young – High Edu: offset	-0,56	0,26	-2,13	0,03
	Old – High Edu: offset	0,29	0,37	0,80	0,43

Table S3. Summary output of the Regression model with visual attention response times as dependent variable (Occipital ROI). The table displays, in the first column, Individual Alpha Peak Frequency (IAPF), exponent, and offset; in the second column, the terms of the mode. The third, fourth, and fifth columns refer to the B (estimate) values, the relative standard error, the t-value associated with the fitted term, and the p-value.

Working Memory		Estimate	Std. Error	t-value	Pr(>|t|)
IAPF	(Intercept)	2,44	2,34	1,04	0,30
	GM_Normalised	-2,90	4,09	-0,71	0,48
	sexM	0,12	0,15	0,79	0,43
	Young – High Edu	-1,34	2,63	-0,51	0,61
	Old – High Edu	-3,35	3,26	-1,03	0,31
	mean_peak_alpha	-0,09	0,12	-0,75	0,46
	Young – High Edu: IAPF	0,00	0,26	0,02	0,99
	Old – High Edu: IAPF	0,29	0,32	0,89	0,38
PW	(Intercept)	-0,18	1,81	-0,10	0,92
	GM_Normalised	-2,16	4,05	-0,53	0,59
	sexM	0,12	0,15	0,80	0,43
	Young – High Edu	1,02	0,63	1,63	0,11
	Old – High Edu	2,22	0,79	2,83	0,01
	mean_power	0,10	0,38	0,25	0,80
	Young – High Edu: PW	0,14	0,45	0,31	0,76
	Old – High Edu: PW	-1,00	0,56	-1,80	0,07
exponent	(Intercept)	-1,36	1,88	-0,72	0,47
	GM_Normalised	-1,41	4,09	-0,35	0,73
	sexM	0,10	0,15	0,66	0,51
	Young – High Edu	1,90	0,76	2,52	0,01
	Old – High Edu	3,68	1,40	2,63	0,01
	mean_exponent	0,61	0,35	1,74	0,08
	Young – High Edu: exponent	-0,47	0,42	-1,13	0,26
	Old – High Edu: exponent	-1,72	0,82	-2,09	0,04
offset	(Intercept)	8,40	4,77	1,76	0,08
	GM_Normalised	-1,97	4,09	-0,48	0,63
	sexM	0,15	0,15	0,99	0,32

**Working Memory**		Estimate	Std. Error	t-value	Pr(>|t|)
	Young – High Edu	-4,40	5,12	-0,86	0,39
	Old – High Edu	-11,79	8,21	-1,44	0,15
	mean_offset	0,53	0,28	1,87	0,06
	Young – High Edu: offset	-0,34	0,32	-1,08	0,28
	Old – High Edu: offset	-0,78	0,51	-1,53	0,13

Table S4. The summary output of the Regression model with working memory accuracy as a dependent variable (Occipital ROI). The table displays, in the first column, Individual Alpha Peak Frequency (IAPF), exponent, and offset; in the second column, the terms of the mode. The third, fourth, and fifth columns refer to the B (estimate) values, the relative standard error, the t-value associated with the fitted term, and the p-value.

Delayed Memory recall		Estimate	Std. Error	t-value	Pr(>|t|)
IAPF	(Intercept)	-0,55	3,92	-0,14	0,89
	GM_Normalised	-0,52	0,15	-3,53	< 0.001
	sexM	0,84	2,52	0,33	0,74
	Young – High Edu	0,79	3,64	0,22	0,83
	Old – High Edu	-0,05	0,22	-0,22	0,83
	mean_peak_alpha	0,02	0,25	0,08	0,94
	Young – High Edu: IAPF	-0,09	0,36	-0,26	0,79
	Old – High Edu: IAPF	0.11	0.31	0.37	0.71
PW	(Intercept)	-0,17	1,72	-0,10	0,92
	GM_Normalised	0,18	3,84	0,05	0,96
	sexM	-0,50	0,14	-3,53	0,01
	Young – High Edu	0,61	0,59	1,03	0,31
	Old – High Edu	1,13	0,75	1,52	0,13
	mean_power	-0,20	0,36	-0,56	0,58
	Young – High Edu: PW	0,29	0,42	0,69	0,49
	Old – High Edu: PW	-0,89	0,53	-1,68	0,10
exponent	(Intercept)	-0,23	1,82	-0,13	0,90
	GM_Normalised	-0,77	3,97	-0,19	0,85
	sexM	-0,50	0,14	-3,45	<0.001
	Young – High Edu	1,25	0,73	1,71	0,09
	Old – High Edu	-0,81	1,36	-0,60	0,55
	mean_exponent	0,13	0,34	0,37	0,71
	Young – High Edu: exponent	-0,14	0,40	-0,34	0,73
	Old – High Edu: exponent	0,38	0,80	0,48	0,63
offset	(Intercept)	-0,01	3,96	0,00	1,00
	GM_Normalised	-0,49	0,14	-3,39	<0.001
	sexM	-2,06	4,96	-0,42	0,68

Delayed Memory recall		Estimate	Std. Error	t-value	Pr(>|t|)
	Young – High Edu	-6,43	7,95	-0,81	0,42
	Old – High Edu	0,23	0,27	0,83	0,41
	mean_offset	-0,19	0,31	-0,61	0,54
	Young – High Edu: offset	-0,39	0,49	-0,79	0,43
	Old – High Edu: offset	-0,55	3,92	-0,14	0,89

Table S5. Summary output of the Regression model with delayed memory accuracy times as dependent variable (Occipital ROI). The table displays, in the first column, Individual Alpha Peak Frequency (IAPF), exponent, and offset; in the second column, the terms of the mode. The third, fourth, and fifth columns refer to the B (estimate) values, the relative standard error, the t-value associated with the fitted term, and the p-value.

ADDITIONAL REGRESSION ANALYSES
FRONTAL BRAIN REGION

Processing Speed: Alertness		Estimate	Std. Error	t-value	Pr(>|t|)
IAPF	(Intercept)	4,02	2,88	1,40	0,16
	GM_Normalised	-4,69	3,95	-1,19	0,24
	sexM	-0,11	0,14	-0,80	0,43
	Young – High Edu	-1,86	3,09	-0,60	0,55
	Old – High Edu	-2,88	2,50	-1,15	0,25
	mean_peak_alpha	-0,11	0,23	-0,50	0,62
	Young – High Edu: IAPF	0,17	0,32	0,54	0,59
	Old – High Edu: IAPF	0,19	0,25	0,77	0,44
PW	(Intercept)	3,49	1,65	2,12	0,04
	GM_Normalised	-3,68	3,91	-0,94	0,35
	sexM	-0,09	0,14	-0,66	0,51
	Young – High Edu	-1,57	0,61	-2,59	0,01
	Old – High Edu	-2,02	0,58	-3,48	<0,01
	mean_power	-1,43	0,61	-2,36	0,01
	Young – High Edu: PW	2,00	0,83	2,40	0,01
	Old – High Edu: PW	1,39	0,69	2,01	0,05
exponent	(Intercept)	2,75	1,69	1,63	0,11
	GM_Normalised	-5,75	3,95	-1,45	0,15
	sexM	-0,11	0,14	-0,75	0,45
	Young – High Edu	-0,76	0,97	-0,79	0,43
	Old – High Edu	-0,93	0,94	-0,98	0,33
	mean_exponent	0,39	0,52	0,74	0,46

Processing Speed: Alertness		Estimate	Std. Error	t-value	Pr(>|t|)
	Young – High Edu: exponent	0,45	0,66	0,69	0,49
	Old – High Edu: exponent	-0,06	0,57	-0,10	0,92
offset	(Intercept)	6,43	7,23	0,89	0,38
	GM_Normalised	-5,17	3,99	-1,30	0,20
	sexM	-0,07	0,15	-0,47	0,64
	Young – High Edu	3,65	8,89	0,41	0,68
	Old – High Edu	-2,17	7,27	-0,30	0,77
	mean_offset	0,20	0,40	0,50	0,62
	Young – High Edu: offset	0,23	0,53	0,43	0,67
	Old – High Edu: offset	-0,07	0,44	-0,16	0,87

Table S6. Summary output of the Regression model with alertness response times as dependent variable (Frontal ROI). The table displays, in the first column, Individual Alpha Peak Frequency (IAPF), exponent, and offset; in the second column, the terms of the mode. The third, fourth, and fifth columns refer to the B (estimate) values, the relative standard error, the t-value associated with the fitted term, and the p-value.

**Processing Speed: Visual Attention**		Estimate	Std. Error	t-value	Pr(>|t|)
IAPF	(Intercept)	3,54	2,49	1,42	0,16
	GM_Normalised	-0,15	3,42	-0,04	0,97
	sexM	0,18	0,12	1,45	0,15
	Young – High Edu H	-1,11	2,67	-0,42	0,68
	Old – High Edu	-3,56	2,17	-1,64	0,10
	mean_peak_alpha	-0,27	0,20	-1,36	0,18
	Young – High Edu: IAPF	0,12	0,27	0,45	0,66
	Old – High Edu: IAPF	0,22	0,22	1,03	0,31
PW	(Intercept)	0,79	1,46	0,54	0,59
	GM_Normalised	0,35	3,46	0,10	0,92
	sexM	0,21	0,13	1,68	0,09
	Young – High Edu	0,01	0,54	0,01	0,99
	Old – High Edu	-1,60	0,51	-3,10	<0,01
	mean_power	-0,19	0,54	-0,36	0,72
	Young – High Edu: PW	0,21	0,74	0,29	0,77
	Old – High Edu: PW	0,30	0,61	0,49	0,62
exponent	(Intercept)	0,40	1,49	0,27	0,79
	GM_Normalised	-0,41	3,50	-0,12	0,91
	sexM	0,21	0,12	1,66	0,10
	Young – High Edu	0,57	0,86	0,66	0,51
	Old – High Edu	-0,61	0,84	-0,73	0,47
	mean_exponent	0,39	0,46	0,83	0,41
	Young – High Edu: exponent	-0,27	0,58	-0,46	0,64
	Old – High Edu: exponent	-0,48	0,50	-0,95	0,34

Processing Speed: Visual Attention		Estimate	Std. Error	t-value	Pr(>|t|)
offset	(Intercept)	4,18	6,27	0,67	0,51
	GM_Normalised	0,00	3,46	0,00	1,00
	sexM	0,16	0,13	1,30	0,19
	Young – High Edu	-11,67	7,70	-1,52	0,13
	Old – High Edu	-6,74	6,30	-1,07	0,29
	mean_offset	0,20	0,35	0,58	0,57
	Young – High Edu: offset	-0,71	0,46	-1,54	0,13
	Old – High Edu: offset	-0,33	0,38	-0,85	0,39

Table S7. Summary output of the Regression model with visual attention response times as dependent variable (Frontal ROI). The table displays, in the first column, Individual Alpha Peak Frequency (IAPF), exponent, and offset; in the second column, the terms of the mode. The third, fourth, and fifth columns refer to the B (estimate) values, the relative standard error, the t-value associated with the fitted term, and the p-value.

Working Memory		Estimate	Std. Error	t-value	Pr(>|t|)
IAPF	(Intercept)	0,91	2,98	0,30	0,76
	GM_Normalised	-3,22	4,09	-0,79	0,43
	sexM	0,10	0,15	0,65	0,51
	Young – High Edu	2,56	3,20	0,80	0,43
	Old – High Edu	1,05	2,60	0,40	0,69
	mean_peak_alpha	0,03	0,24	0,11	0,91
	Young – High Edu: IAPF	-0,35	0,33	-1,08	0,28
	Old – High Edu: IAPF	-0,05	0,26	-0,21	0,84
PW	(Intercept)	1,43	1,73	0,82	0,41
	GM_Normalised	-1,95	4,10	-0,48	0,64
	sexM	0,12	0,15	0,80	0,43
	Young – High Edu	-1,50	0,64	-2,35	0,02
	Old – High Edu	-0,52	0,61	-0,86	0,39
	mean_power	-1,09	0,64	-1,71	0,09
	Young – High Edu: PW	0,92	0,88	1,06	0,29
	Old – High Edu: PW	1,36	0,73	1,88	0,06
exponent	(Intercept)	1,44	1,78	0,81	0,42
	GM_Normalised	-1,93	4,18	-0,46	0,65
	sexM	0,10	0,15	0,67	0,50
	Young – High Edu	-1,55	1,03	-1,51	0,13
	Old – High Edu	-0,74	1,00	-0,74	0,46
	mean_exponent	-0,54	0,55	-0,98	0,33
	Young – High Edu: exponent	0,50	0,69	0,72	0,47
	Old – High Edu: exponent	0,75	0,60	1,25	0,21

Working Memory		Estimate	Std. Error	t-value	Pr(>|t|)
offset	(Intercept)	-5,12	7,44	-0,69	0,49
	GM_Normalised	-2,29	4,11	-0,56	0,58
	sexM	0,15	0,15	1,02	0,31
	Young – High Edu	11,99	9,15	1,31	0,19
	Old – High Edu	12,55	7,48	1,68	0,10
	mean_offset	-0,35	0,41	-0,85	0,40
	Young – High Edu: offset	0,77	0,55	1,40	0,16
	Old – High Edu: offset	0,73	0,45	1,62	0,11

Table S8. The summary output of the Regression model with working memory accuracy as a dependent variable (Frontal ROI). The table displays, in the first column, Individual Alpha Peak Frequency (IAPF), exponent, and offset; in the second column, the terms of the mode. The third, fourth, and fifth columns refer to the B (estimate) values, the relative standard error, the t-value associated with the fitted term, and the p-value.

Delayed Memory recall		Estimate	Std. Error	t-value	Pr(>|t|)
IAPF	(Intercept)	-2,74	2,85	-0,96	0,34
	GM_Normalised	-0,73	3,91	-0,19	0,85
	sexM	-0,50	0,14	-3,47	0,00
	Young – High Edu	1,35	3,06	0,44	0,66
	Old – High Edu	3,64	2,48	1,47	0,14
	mean_peak_alpha	0,26	0,23	1,13	0,26
	Young – High Edu: IAPF	-0,12	0,31	-0,37	0,71
	Old – High Edu: IAPF	-0,25	0,25	-0,99	0,32
PW	(Intercept)	0,16	1,64	0,10	0,92
	GM_Normalised	-0,29	3,89	-0,07	0,94
	sexM	-0,50	0,14	-3,59	<0,01
	Young – High Edu	0,19	0,61	0,31	0,76
	Old – High Edu	0,40	0,58	0,69	0,49
	mean_power	-0,76	0,60	-1,25	0,21
	Young – High Edu: PW	-0,21	0,83	-0,26	0,80
	Old – High Edu: PW	1,11	0,69	1,61	0,11
exponent	(Intercept)	-1,00	1,68	-0,60	0,55
	GM_Normalised	-2,92	3,94	-0,74	0,46
	sexM	-0,52	0,14	-3,70	0,00
	Young – High Edu	1,89	0,97	1,95	0,05
	Old – High Edu	3,39	0,94	3,60	<0,01
	mean_exponent	1,17	0,52	2,24	0,03
	Young – High Edu: exponent	-1,18	0,65	-1,80	0,07
	Old – High Edu: exponent	-1,37	0,57	-2,42	0,66

Delayed Memory recall		Estimate	Std. Error	t-value	Pr(>|t|)
offset	(Intercept)	6,29	7,20	0,87	0,38
	GM_Normalised	-1,84	3,98	-0,46	0,64
	sexM	-0,51	0,15	-3,48	0,01
	Young – High Edu	-3,49	8,85	-0,39	0,69
	Old – High Edu	-5,93	7,24	-0,82	0,41
	mean_offset	0,36	0,40	0,90	0,37
	Young – High Edu: offset	-0,22	0,53	-0,41	0,68
	Old – High Edu: offset	-0,43	0,44	-0,99	0,33

Table S9. Summary output of the Regression model with delayed memory accuracy times as dependent variable (Frontal ROI). The table displays, in the first column, Individual Alpha Peak Frequency (IAPF), exponent, and offset; in the second column, the terms of the mode. The third, fourth, and fifth columns refer to the B (estimate) values, the relative standard error, the t-value associated with the fitted term, and the p-value.

Parcels of the Desikan-Killiany ATLAS aggregated in the ROIs	
Occipital ROI<br>*(averaged across left and right hemispheres)*	Frontal ROI<br>*(averaged across left and right hemispheres)*
Cuneus<br>*Lateral-occipital*<br>*Lingual*<br>*Peri-calcarine*<br>*Precuneus*	*Caudal-middle-frontal; Frontal pole*<br>*Lateral-orbito-frontal; Medial-orbito-frontal*<br>*Paracentral; Pars-opercularis*<br>*Pars-orbitalis; Pars-triangularis*<br>*Pre-central; Rostral-middle-frontal*<br>*Superior-frontal*

Table S10. Desikan-Killiany ATLAS aggregated in the ROIs (Regions of Interest). In the left-hand side column, the parcels of the Occipital ROI are reported; in the right-hand side column, the parcels of the Frontal ROI are reported.
